# Transcription Factors as Novel Therapeutic Targets and Drivers of Prostate Cancer Progression

**DOI:** 10.3389/fonc.2022.854151

**Published:** 2022-04-25

**Authors:** Kangzhe Xie, Keely Tan, Matthew J. Naylor

**Affiliations:** Charles Perkins Centre, School of Medical Sciences, Faculty of Medicine & Health, University of Sydney, Sydney, NSW, Australia

**Keywords:** prostate cancer, transcription factor, epigenetic, ubiquitin-proteasome system, protein-protein interactions, targeting approaches

## Abstract

Prostate cancer is the second most diagnosed cancer among men worldwide. Androgen deprivation therapy, the most common targeted therapeutic option, is circumvented as prostate cancer progresses from androgen dependent to castrate-resistant disease. Whilst the nuclear receptor transcription factor, androgen receptor, drives the growth of prostate tumor during initial stage of the disease, androgen resistance is associated with poorly differentiated prostate cancer. In the recent years, increased research has highlighted the aberrant transcriptional activities of a small number of transcription factors. Along with androgen receptors, dysregulation of these transcription factors contributes to both the poorly differentiated phenotypes of prostate cancer cells and the initiation and progression of prostate carcinoma. As master regulators of cell fate decisions, these transcription factors may provide opportunity for the development of novel therapeutic targets for the management of prostate cancer. Whilst some transcriptional regulators have previously been notoriously difficult to directly target, technological advances offer potential for the indirect therapeutic targeting of these transcription factors and the capacity to reprogram cancer cell phenotype. This mini review will discuss how recent advances in our understanding of transcriptional regulators and material science pave the way to utilize these regulatory molecules as therapeutic targets in prostate cancer.

## Introduction

Prostate cancer is the second most diagnosed cancer in men worldwide, with approximately 1.4 million cases in 2020 alone (1). Prostatic intraepithelial neoplasia (PIN) is a premalignant lesion characterized by the uncontrollable cell growth within the prostate gland (2). This unchecked proliferation precedes the development of localized prostate adenocarcinoma, whereby the tumor increases in volume and cells begin to infiltrate through the basement membrane. The initial pathogenesis of this disease is largely dependent on the activity of the transcriptional factor, androgen receptor (AR) (3). However, once the disease progresses to a more aggressive phenotype, the tumor becomes androgen resistant, evolving into castrate-resistant prostate carcinoma (CRPC) (4). Metastatic CRPC (mCRPC) is the advanced/final stage of the disease, with cancer cells undergoing metastasis to distal organs such as bone, liver and lungs (5). In addition to androgen resistance, phenotypic changes such as alteration to chromatin structure and nucleus enlargement also occur during the malignant transformation in prostate cells (6, 7).

**Figure 1:**
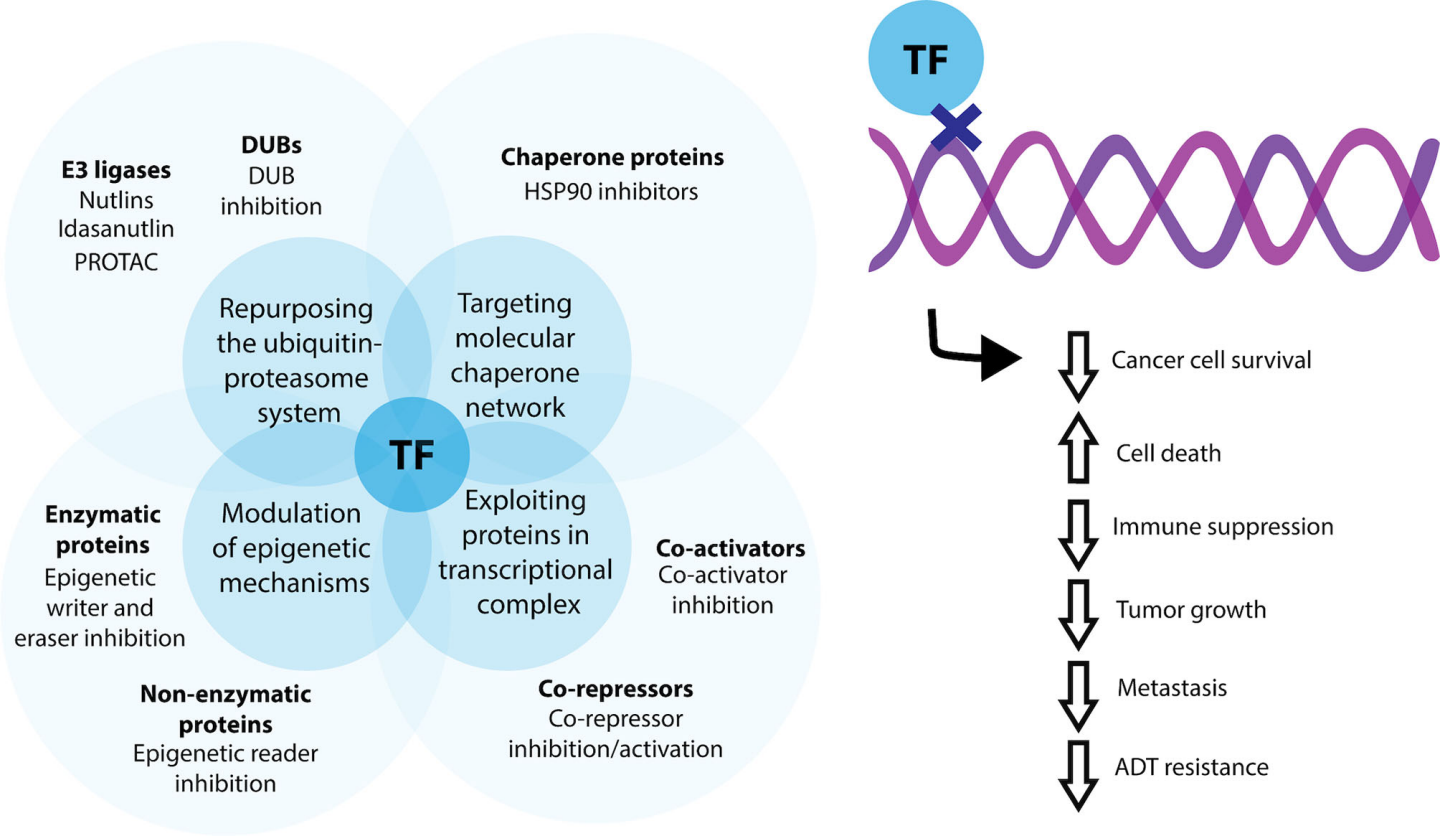
Current indirect methods for targeting transcription factors for prostate cancer therapy. Transcription factor (TF) activity may be indirectly modulated by targeting enzymatic and non-enzymatic proteins involved in epigenetic signaling, through repurposing and manipulating aspects of the ubiquitin-proteasomal system for control of transcription factor degradation, by targeting the molecular chaperone network and by exploiting co-activator and co-repressors associated with a transcriptional complex. Using these methods, transcription factor activity can be favorable modified to decrease cancer cell survival, overall tumour growth and the potential for metastatic dissemination.

AR is a ligand activated transcription factor and it functions through the binding of androgens, such as testosterone and 5-α-dihydrotestosterone, which releases AR from its chaperone heat shock protein (HSP) 90. Similar to many steroid hormone nuclear receptors, this results in translocation of AR to the nucleus to regulate the expression of genes associated with growth and maintenance of the prostate epithelium (8). AR, along with a small number of other transcription factors, have been well established as regulatory molecules that govern prostate cell phenotype and are implicated in the initiation and progression of prostate cancer (**Table 1**, also see reviews (2, 36). As the same transcription factor has the ability to bind to regulatory regions of different genes (37), targeting transcription factors provides a direct target in developing effective treatments for prostate cancer and allows for the coordinated inhibition of various oncogenic genes and signaling pathways. Whilst several direct approaches to alter transcription factor expression such as siRNA loaded nanoparticles and lentiviruses are under development, this mini review will focus on indirect approaches such as modulation of epigenetic mechanisms, manipulation of the ubiquitin-proteasome system, targeting the molecular chaperone network and exploitation of proteins in transcriptional complexes, with the discussion of some recent successful attempts (**Figure 1**).

**Table 1:** Identified transcription factor targets and their implications in prostate cancer.

Target Types	Transcription Factors/Proteins	Biological Functions & Implications in Prostate Cancer	References
Nuclear hormone receptors	AR	Drives prostate cancer cell proliferation; maintain prostate cancer cell survival; mutation and amplification of AR in prostate cancer contributes to androgen deprivation therapy resistance.	(3)
	ERs	ERα stimulates prostate cancer cell proliferation and promotes the development of prostate malignancy; ERβ downregulates AR signaling and acts as tumour suppressor.	(9)
	Glucocorticoid receptor	Promotes prostate cancer cell proliferation; contributes to androgen deprivation therapy resistance.	(10)
	Progesterone receptor	Prevents prostate cancer cell migration and invasion.	(10)
	Vitamin D receptor	Promotes cell differentiation and apoptosis; inhibits cell growth, prostate cancer cell migration and angiogenesis.	(10)
	Retinoic acid receptors	Suppresses AR signaling; reduces prostate cancer cell proliferation.	(10)
	ERRα	Regulates energy homeostasis in prostate cells; regulates prostate cancer cell proliferation	(11)
Tumour protein p53	p53	Responds to cellular stress; regulates the expression of genes that are involved in DNA repair, cell arrests and apoptosis; inactivation of p53 is associated with poor clinical outcome.	(12)
ETS fusions	TMPRSS2-ERG fusion	Common chromosomal translocation observed in prostate cancer; increases incidence of prostatic intraepithelial neoplasia development.	(13–15)
Histone methyltransferase	EZH2	Acts as transcription regulators for genes such as PD-1; often overly expressed in advanced stage of prostate cancer.	(16)
MYC	c-Myc	Remodels chromatin structures to stimulate prostate cancer cell growth; promotes oncogenic signaling *via* hyperacetylation.	(17, 18)
	n-Myc	Maintains prostate tumour cell survival; promotes poorly differentiated aggressive prostate cancer phenotype; drives the development of neuroendocrine prostate cancer.	(19)
BET proteins	BRD2	Regulates by androgen; interacts with YY1 to co-activate downstream oncogenic genes; promotes prostate cancer cell growth.	(20)
	BRD4	Regulates the expression of oncogenic transcription factor MYC; regulates prostate cancer cell proliferation; drives ETM transition in CRPC.	(21, 22)
Ubiquitin-proteasome system	MDM2	Regulates prostate cancer cell growth, apoptosis, and the expression of tumour suppressor p53.	(23–25)
	USP2a	Regulates the expression of p53 indirectly by deubiquitinating MDM2.	(26)
	USP5	Acts as a DUB for p53; regulates the expression of p53.	(27)
	USP9X	Acts as DUB for ERG; regulates the expression of transcription factor ERG.	(28)
Core binding factor transcription complex	RUNX proteins	Promotes prostate cancer cell growth and increases metastatic potential *via* matrix metalloproteinase signaling.	(24)
Molecular chaperone	HSP90	Interacts with oncogenic transcription factors include AR, p53 and HIF-1α	(29, 30)
Hypoxia inducible factor transcription complex	HIF-1α	Induces angiogenesis; promotes cancer cell proliferation and survival; facilitates the development of CRPC and metastasis	(31)
Tumour suppressing phosphatase	PTEN	Regulates the PI3K-Akt signaling pathway; loss of PTEN increases the aggressiveness of prostate cancer.	(32)
Prostate specific homeobox gene	NKX3.1	Regulates prostate epithelial cells differentiation and growth; reduced level of NKX3.1 increases the aggressiveness of prostate cancer.	(33)
NF- κB	NF- κB	Promotes prostate tumour invasion; increases metastatic potential; inhibits prostate cancer cell death; contributes to chemotherapy resistance.	(34)
FOX protein family	FOXA1	Drives prostate cancer cell proliferation; maintain prostate cancer cell survival; regulates ETM transition.	(35)

AR, androgen receptors; ER, estrogen receptor; ERRα, estrogen related receptor alpha ETS, E-twenty-six; TMPRSS2, transmembrane-protease-serine 2; ERG, ETS related gene; EZH2, enhancer of zeste homolog 2; PD-1, programmed cell death protein 1; BET, bromodomain extra-terminal enhancer; BRD, bromodomain-containing protein; YY1, transcription factor Ying Yang 1; ETM, epithelial to mesenchymal; CRPC, castrate resistant prostate cancer; DUB, deubiquitinase; MDM2, murine double minute 2; USP, ubiquitin-specific peptidase; RUNX, runt-related transcription factor; HSP, heat shock protein; HIF-1α, hypoxia inducible factor 1 alpha; PTEN, phosphatase and tensin homolog; PI3K, phosphoinositide-3-kinase; Akt, protein kinase B; NF- κB, nuclear factor kappa B; FOX, Forkhead box.

## Therapeutic Targeting of Transcription Factors

Historically, many transcription factors have been considered as ‘undruggable’ targets, owing to their ‘intrinsically disordered’ interaction network formed with their functional partners (38, 39). In cells, transcription factors regulate gene expression through protein-protein interactions (PPIs) with their co-activators and co-repressors as well as *via* direct sequence specific DNA binding (40). As a result, the lack of enzymatic activities and catalytic sites presents a major blockade in the development of transcription factor inhibitors and modulators (41). In addition, the transcription factor-DNA binding interfaces are often positively charged and structurally convex, whereas the sites for transcription factor-co-regulator interactions are much flatter than the typical enzyme ligand binding pockets (42, 43). Together, these properties further exacerbate the challenges in developing small molecule inhibitors and modulators with desirable ADME (Absorption, Distribution, Metabolism and Excretion) indices (42, 43). In recent years, a plethora of studies have demonstrated success in targeting transcription factors in prostate cancer, showcasing the feasibility of this approach, and challenging previous dogma. In particular, chemical inhibitors targeting the AR ligand binding domains such as bicalutamide and enzalutamide, have been developed (44, 45). Whilst the use of AR inhibitors are now amongst the primary options for androgen-targeted therapies in early-stage prostate cancers (45, 46), these options become ineffective once the tumor becomes castrate-resistant, as they are able to circumvent androgen targeted treatments *via* various mechanisms which include AR amplification, point mutation, splicing variants and replacing AR functions with glucocorticoid receptors (4, 47, 48). As there is a profound paucity of effective treatments for mCRPC patients (49), research into methods of targeting non-AR transcription factors in prostate cancer is critical.

## Modulation of Epigenetic Mechanisms

Modulating epigenetic signaling pathways is one approach used to target oncogenic transcription factors within prostate cancer. As enzymes are druggable targets with relatively high tractability, epigenetic writers and erasers such as acetyl transferase, methyltransferase, deacetylases and demethylases provide a direct target for inhibitor development (50). Enhancer of zeste homolog 2 (EZH2) is an important epigenetic regulator and in prostate cancer, it was found that EZH2 negatively regulates the expression of interferon stimulated genes (ISGs) such as programmed cell death protein 1 (PD-1) and major histocompatibility complex, creating an immunosuppressive tumor microenvironment and increasing resistance to immune checkpoint blockade (ICB) therapies (16). In a recent study by Morel and colleagues, inhibition of EZH2 restored the expression of ISGs and reversed the resistance to ICB treatments, highlighting the therapeutic potential of EZH2 inhibitors in prostate cancer (51). The clinical applicability of EZH2 inhibitors was reinforced by Bai and colleagues, where EZH2 inhibition with GSK126 prevented prostate specific antigen expression and overcame enzalutamide resistance in CRPC (52). Furthermore, the development of neuroendocrine prostate cancer (NEPC), an aggressive subtype of CRPC, was also found to be associated with dysfunctional EZH2 activity (53). Using GSK126, Dardenne and colleagues showed that NEPC cells were more sensitive to EZH2 inhibition than androgen sensitive LNCaP cells (54), however, the clinical efficacy of EZH2 inhibitors against NEPC warrants further investigation. Whilst EZH2 represents a major target for prostate cancer, alternative targets include histone acetyl transferase E1A binding protein (p300) and CREB binding protein (CBP). In prostate cancer, p300 and CBP interact with numerous oncogenic transcription factors, including p53, MYC and AR, to drive tumour progression (55). These epigenetic enzymes can be inhibited by a CellCentric developed compound, CCS1477, where administration of CCS1477 was shown to downregulate the expression of AR and MYC, resulting in decreased tumor growth in a 22Rv1 xenograft model of CRPC (56, 57).

Alternatively, targeting regulators that do not possess enzymatic activities in the epigenetic signaling pathway have also proven successful. MYC is one of the most dysregulated transcription factors in human cancers (17). In prostate cancer, MYC remodels the chromatin structure to stimulate cell growth and promote oncogenic signaling *via* hyperacetylation (17, 18) and it has been shown that these oncogenic effects are partly mediated by the epigenetic reader protein, bromodomain extra-terminal (BET) (58). As a result, targeting the BET family of proteins provides a potential avenue to indirectly regulate the expression of MYC, ultimately regressing prostate tumor progression. Indeed, I-BET762, a BET inhibitor, has been shown to reduce MYC expression. This was associated with decreased prostate cancer cell proliferation, increased programmed cell death and reduced *in vivo* prostate tumour burden, highlighting the possibility of targeting BET proteins as a treatment for prostate cancer (59). The therapeutic potential of BET inhibition is further accentuated by JQ1, an inhibitor that targets bromodomain containing protein (BRD) 4 (60). BRD4 is a member of the BET family of proteins, and it has been shown to interact with transcription factors such as AR and MYC to mediate oncogenic effects (21). More recently, it has been suggested that BRD4 also plays a role in regulating tumor immune microenvironments. This is supported by Mao and colleagues, where BRD4 inhibition with JQ1 reduced PD-1 expression and promoted CD8-mediated lysis of prostate tumor cells both *in vitro* and *in vivo* (61). In addition, JQ1 was found to mediate anticancer effect by downregulating the expression of Achaete-scute homolog 1 (ASCL1) in small-cell lung cancer (62). With recent evidence suggesting that ASCL1 as a key driver for NEPC (63), JQ1 along with other BET inhibitors may have potential as NEPC therapeutics. The BET inhibitors, BMS-986158 and RO6870810 are now also in various phases of multicancer clinical trials, with the pan-BET inhibitor ZEN-3694, showcasing therapeutic efficacy in a Phase Ib/IIa mCRPC study (64–66).

## Manipulation of the Ubiquitin-Proteasome System

Another way to target transcription factor in prostate cancer is utilizing the ubiquitin-proteasome system (UPS). Appropriate levels of transcription factor expression in cells is key in maintaining cellular homeostasis (67). Aberrant transcription factor expression or failure in the expression of regulatory circuits may lead to catastrophic effects and result in pathophysiological states. Ubiquitin ligases (E3 ligases) are enzymes in the UPS that catalyze the cellular process of ubiquitylation, in which ubiquitin covalently attaches to the substrate protein for proteasomal degradation as a method to regulate transcription factor expression (68, 69). This unique degradation pathway provides a potential platform for controlling transcription factor expression in prostate cancer.

In several human cancers, including prostate cancer, the expression of transcription factor and tumor suppressor p53, is known to be highly dysregulated (70). This dysregulation can arise from the increased activity of murine double minute 2 (MDM2), an E3 ligase, which decreases the expression of p53 and ultimately results in poor clinical outcomes for the patient (12, 23). Thus Nutlins, a novel class of MDM2 inhibitors, were developed by Vassilev and colleagues. The use of these inhibitors increased cellular expression of p53 and its target gene p21 (71), whilst further research using *in vivo* mouse xenograft models with androgen dependent LNCaP and androgen independent 22Rv1 cell lines, demonstrated increased apoptosis and reduced tumor burden in both cell types following Nutlins treatment (72, 73). To further improve the potency and selectivity of these Nutlins, a second-generation compound Idasanutlin (RG7738/RO5503781), was developed (74). Research shows that use of this compound induces cell death *via* a combined mechanism of cell cycle arrest and cytotoxic insult in LNCaP cells (75). The clinical applicability of E3 ligase inhibition has recently been assessed in a Phase I clinical trial for prostate cancer patients who have not received docetaxel treatment previously (76). Whilst the trial was terminated due to safety concerns and financial withdrawal from Roche, promising preliminary results highlight the potential for this class of compound, and E3 ligases in general, to be further investigated as a prostate cancer therapy.

The utilization of the UPS (more specifically E3 ligase) to target transcription factors within prostate cancer was taken further with the discovery of proteolysis targeting chimeras (PROTACs). PROTACs are bifunctional molecules comprised of a protein interacting ligand, as well as an E3 ligase recruiting ligand (77). The two ligands in PROTACs are linked in a covalent manner, with protein interacting ligand binding with the protein of interest such as a transcription factor, whereas E3 ligase recruiting ligand facilitates the process of ubiquitylation and subsequent protein degradation (78). PROTACs generate a ‘knocked-down’ effect in cells, abrogating the cellular function of the protein of interest (79). Furthermore, it was discovered that this process is highly catalytic, where a single PROTAC molecule can eliminate multiple protein of interest (80). As discussed above, BET proteins regulate the expression of many pro-oncogenic transcription factors such as AR and MYC, and pharmacological inhibition of these regulatory molecules results in an anticancer effect. Therefore, the use of PROTACs could represent another avenue of pharmacological modification on dysregulated transcription factor expression in prostate cancer. WWL0245 is a highly selective and potent PROTAC-based degrader of BRD4 and has been shown to function by inducing cell cycle arrest of the androgen sensitive prostate cancer cell lines, LNCaP and VCaP, *in vitro*. This was simultaneously associated with the downregulation of oncogenic transcription factors AR and c-Myc, which highlights the therapeutic potential and clinical feasibility of this approach in prostate cancer (81). Such notion was further supported by Raina and colleagues, where they demonstrated that pan-BET PROTAC, ARV-771, induced cell apoptosis and tumour regression in a mouse xenograft model of CRPC (82). Excluding BET, most of the current research is focused on the PROTAC-based approached on targeting AR (83–85), whereas attempts to target other dysregulated transcription factors in prostate cancer *via* PROTACs is limited. Thus, identifying a wider variety of protein interacting ligands should be the topic of prospective research.

Another way to alter transcription factor expression is to inhibit the activities of deubiquitinases (DUBs). DUBs are enzymes that remove ubiquitin proteins in the UPS, terminating the ubiquitin-mediated proteasomal degradation process (86). Ubiquitin-specific peptidase (USP) 2a act as a DUB for MDM2, in which it positively regulates the expression of MDM2 (26). Since MDM2 is an E3 ligase for transcription factor p53, inhibition of USP2a would promote the proteasomal degradation of MDM2 and indirectly regulate the expression of p53 (23, 86). This was supported by Stevenson and colleagues, where siRNA inhibition of USP2a led to the accumulation of p53 protein *in vivo*, highlighting the therapeutic potential to inhibit DUBs in prostate cancer (87). Inhibition of DUBs in the context of prostate cancer is not limited to the p53 signaling pathway. The gene fusion product transmembrane-protease-serine-2 (TMPRSS2)-ETS-related gene (ERG) as a result of chromosomal translocation is observed in 20 to 50% of prostate cancer patients with different ethnicities (13, 14). The TMPRSS2-ERG fusion protein was showed to mediate prostate cancer cell invasion and activation of transcriptional programs for invasion-associated genes (15). The expression of ERG is regulated by E3 ligase tripartite-motif-containing-25 (TRIM25), whereas USP9X acts as DUB for ERG deubiquitylation (28, 88). Thus, target inhibition of ERG or TMPRSS2 may be beneficial in prostate cancer. WP1130 is a small molecule inhibitor comprised of two protein reactive moieties, a 2-bromo-pyridine functional group as well as an α,β-unsaturated amide moiety that is able to undergo Michael addition reactions (89). This enables WP1130 to interact with proteins in a partly selective manner and exerts inhibitory effects on multiple DUBs such as USP5, USP9X and USP14 (90). It has been shown that WP1130 reduces the level of ERG *in vitro* by inhibiting the enzymatic function of USP9X. This was associated with a decrease in tumour volume in murine xenografts with VCaP cells, highlighting the clinical feasibility to target DUBs in prostate cancer (28).

## Targeting the Molecular Chaperone Network

The molecular chaperone network is responsible for various biological processes such as appropriate protein folding, intracellular localization, and degradation, thus maintaining protein homeostasis in cells (29). Chaperone protein such as HSP90 exert these functions by interacting with a diverse range of client proteins, and amongst them, many are oncogenic transcription factors. They include AR, p53 and hypoxia inducible factor (HIF)-1α (29, 30). As a result, disrupting HSP90-transcription factor interactions *via* small molecule inhibitors provide a potential pathway to rectify the dysregulated mechanisms that cause prostate malignancy. 17-AAG is the first-in-class HSP90 inhibitor developed by Schnur and colleagues. However, the weak potency and poor bioavailability of this compound has sparked further optimization (30). Ganetespib is a second-generation HSP90 inhibitor with improved potency. It has been shown that Ganetespib induced cell cycle arrest in LNCaP and LAPC4 cells and resulted in tumor regression in a PDX model of CRPC (91), highlighting the feasibility and clinical applicability of HSP90 inhibition as an anticancer treatment. This notion was reinforced recently by SU086, another novel HSP90 inhibitor that was found to reduce the proliferation of PC3 and DU145 prostate cancer cells *in vitro* and inhibit tumor growth in a preclinical murine model of prostate cancer (92). In addition to HSP90, other emerging targets from the molecular chaperone network include HSP70 and HSP90 co-chaperone CDC37, however, drug-like inhibitors targeting these two proteins are yet to be developed (93). Whilst targeting chaperone proteins other than HSP90 in prostate cancer are not well understood and requires further investigation, it represents a novel strategy for prostate cancer treatment.

## Exploiting Other Proteins in a Transcriptional Complex

Another approach to modulate transcription factor expressions can be achieved *via* the exploitation of proteins in a transcriptional complex. HIF is a transcriptional complex that plays a key role in inducing angiogenesis, an essential requirement for prostate tumour growth and the CRPC development. Prior to sufficient vascular development by the prostate tumour, cancer cells must adapt to the low oxygen concentration to fulfil their large energy expenditure (31). Estrogen related receptor alpha (ERRα) is involved in the regulation of prostate energy homeostasis (11). It has been shown that ERRα can interact with hypoxia inducible factor 1 (HIF) transcription factor complex to prevent HIF-1α from undergoing proteasomal degradation and augments the cellular adaptive response to hypoxia generated by the prostate tumour cells (94, 95). As a result, interference of this indispensable PPI is a lucrative approach to develop prostate cancer therapeutics. XCT790 is an inverse agonist of ERRα (94). It was demonstrated that administration of XCT790 attenuated ERRα-HIF-1 interactions and reduced the expressions of HIF-1 (95). This was associated with a decrease in LNCaP prostate cancer cell proliferation *in vitro* (95), outlining the clinical applicability of this approach to disrupt transcription factor interactions.

The heterodimeric transcription factor complex core binding factor (CBF) is another emerging target. CBF consists of two proteins: DNA binding runt-related transcription factor (RUNX) and its non-DNA binding beta subunit (CBFβ) (96). The CBFβ functions as a co-activator to RUNX, resulting in RUNX being relieved from its autoinhibited state, facilitating the CBF complex binding to DNA and regulation of target gene expression (97). In recent years, there has been growing recognition of RUNX transcription factors in promoting cell growth and metastatic potential of prostate cancer *via* matrix metalloproteinase signaling (24). Therefore, targeting such essential PPIs may disrupt the transcription process of oncogenic genes, resulting the anticarcinogenic effects. Successful targeting of this modality was achieved using a monovalent derivative of the AI-10-49 scaffolds, a bivalent inhibitor that was originally developed to target CBFβ-smooth muscle myosin heavy chain interactions (98). This novel monovalent inhibitor interferes the binding between wildtype CBFβ and the RUNX1 protein by altering their conformational dynamics (99). In a study on triple negative breast cancer, CBFβ-RUNX1 inhibition was shown to abolish colony formation and alter the expression of epithelial-mesenchymal transition genes, a characteristic cancer hallmark associated with metastasis (99). With regards to prostate cancer, this finding highlights the therapeutic potential to disrupt RUNX interaction circuity, which may be applicable for developing prostate malignancy therapeutics.

## Conclusion

This mini review briefly summarized the recent success in targeting non-AR transcription factors. However, it is worth noting that possible approaches to modulate non-AR transcription factors are not restricted to the ones mentioned above, and these successful discoveries only mark the starting point of further transcription factor research. Nevertheless, the newly discovered inhibitors and modulators represent an encouraging potential to develop effective treatment options for mCRPC by targeting non-AR transcription factor.

## Author Contributions

KX designed the study, was responsible for writing the article and the creation of the table. KT was responsible for writing the article and the creation of the figure. MN designed the study and was responsible for writing and revising the manuscript. All authors contributed to the generation of the concepts and ideas provided. All authors contributed to the article and approved the submitted version.

## Funding

This work was supported by Cancer Council NSW Research Project Grant (RG 20-08) and Priority-driven Collaborative Cancer Research Scheme (Grant #1130499), funded by the National Breast Cancer Foundation Australia with the assistance of Cancer Australia awarded to MN.

## Conflict of Interest

The authors declare that the research was conducted in the absence of any commercial or financial relationships that could be construed as a potential conflict of interest.

## Publisher’s Note

All claims expressed in this article are solely those of the authors and do not necessarily represent those of their affiliated organizations, or those of the publisher, the editors and the reviewers. Any product that may be evaluated in this article, or claim that may be made by its manufacturer, is not guaranteed or endorsed by the publisher.
